# Living longer or living better - priorities among head and neck cancer survivors

**DOI:** 10.1186/s12885-026-17298-z

**Published:** 2026-11-03

**Authors:** Anna Thomsen, Nourane Trigui, Jonas Rast, Emily Newton-Tanzer, Mireia Gonzalez Donate, Markus Hoffmann, Susanne Wiegand

**Affiliations:** 1 Department of Otorhinolaryngology, Head and Neck Surgery; Phoniatrics and Pediatric Audiology, University Hospital Schleswig-Holstein, Kiel, Germany; 2 Institute of Medical Statistics, Kiel University, University Hospital Schleswig- Holstein, Kiel, Germany

**Keywords:** Head and neck cancer, Quality of life, Length of life, Care planning, Treatment decision

## Abstract

**Background:**

Head and neck cancer and its treatment can profoundly affect physical function, psychosocial well-being, and overall quality of life (QoL). As survival rates improve, integrating patient-reported outcomes and individual treatment preferences into clinical decision-making has become increasingly important. This study aimed to assess the preferences of head and neck cancer (HNC) survivors regarding QoL versus life prolongation in order to better inform patient-centered treatment decisions.

**Methods:**

Adult head and neck cancer survivors completed validated questionnaires assessing health-related QoL (EORTC QLQ-C30), distress (Distress Thermometer), and preferences for QoL versus life prolongation (QQ questionnaire). Multivariable regression analyses were used to identify predictors of QoL and distress, while associations between patient characteristics and treatment preference were examined using group comparisons.

**Results:**

The study included 207 patients (70.5% male; mean age 66.2 years). The median global health status (QLQ-C30) was 66.6, and the median distress score was 3, indicating a relatively good overall QoL and a comparatively low level of psychological distress. In multivariable analysis, lower distress and employment (both *p* < 0.001) as well as surgical treatment and lower comorbidity (both *p* < 0.05) were independently associated with better QoL. Independent predictors of distress included sex, global health status, tumor stage, educational level, and treatment modality (all *p* < 0.05). Regarding preferences (*n* = 202), 53.0% of patients prioritized QoL, 21.8% favored life prolongation, and 25.2% reported no clear preference. Preference was significantly associated with age (*p* = 0.035), with older patients favoring QoL, and with tumor stage (*p* = 0.032), with the differences between options diminishing in advanced stages.

**Conclusion:**

QoL in head and neck cancer survivors is shaped by both clinical and psychosocial factors. Lower distress, employment, surgical treatment, and lower comorbidity were associated with better QoL, while most patients prioritized QoL over life prolongation, particularly at older age. These findings emphasize the need to align treatment strategies with patient values, placing QoL at the center of clinical decision-making.

## Introduction

Fortunately, the prognosis of head and neck cancer (HNC) has improved over the past few decades as reflected by an increase in the five-year survival rate across all subtypes [1]. This improvement is partly due to improved treatment methods [2]. However, patients with head and neck tumors face particular challenges, as the disease often has serious effects on essential aspects of life, such as breathing, speech, eating, and social interaction. These challenges are further compounded by the complexity and intensity of the multimodal treatment approaches commonly used in head and neck oncology, including surgery, radiotherapy, and systemic therapies. While such combinations can improve tumor control, they often increase the risk of cumulative toxicities and long-term side effects [3, 4]. As a result, patients frequently experience persistent impairments that extend beyond the acute treatment phase and require ongoing medical and supportive care.

Both the cancer itself and its treatment therefore can profoundly impair health-related QoL [5]. Particularly in this patient group, there is often a conflict between prolonging life and maintaining QoL, as the therapies used are frequently associated with significant side effects that negatively affect daily functioning, emotional well-being, and social participation. It has been shown that the loss of swallowing, speaking, and breathing functions, as well as the consequences of tracheostomy, PEG feeding, or permanent functional limitations, trigger not only physical but also psychosocial stressors, which can lead patients to weigh the burden of their illness more heavily than the fear of early death [6]. This highlights the importance of not only evaluating clinical outcomes but also considering the broader, long-term consequences of treatment from the patient’s perspective.

In oncology, recent decades have seen a shift from a sole focus on prolonging life to a more patient-centered treatment approach. In addition to maximizing survival, clinical decision-making is increasingly focused on maintaining and improving health-related QoL. With prognoses continuing to improve and treatment methods becoming less invasive, the question of how patients want to live their lives after cancer treatment has come to the forefront.

Many previous studies have examined the impact of common treatment methods on perceived QoL without identifying a clear favorite, although there is a tendency toward surgery [7]. However, the range of treatment options is continually expanding; for example, the use of checkpoint inhibitors is currently evolving [8]. The benefits patients derive from these ongoing innovations motivate further improvement of treatment approaches.

Nevertheless, further research is needed to better understand the individual trade-offs between lifespan and QoL in the daily oncology setting for patients with HNC and to derive patient-centered treatment strategies from these insights. Therefore, this study aims to assess the preferences of patients with head and neck tumors regarding QoL versus length of life (LL).

## Patients and methods

### Study sample

Adult head and neck cancer survivors were recruited from the aftercare program of the Department of Otorhinolaryngology. All participants provided written informed consent. Participants were excluded if they had insufficient proficiency in German, had cognitive impairments, or were unable to provide informed consent. The study was approved by the institutional review board of the University (vote: D 496/25). In total, 207 patients consented to participate.

Sociodemographic, clinical and disease-related data were extracted from the patients’ records. In addition, health-related QoL, cancer-related psychosocial distress and cancer patients’ preferences regarding quality (QoL) versus length of life (LL) were assessed using the following validated instruments. Distress and quality of life data were available for 207 patients. Complete responses to the QQ questionnaire were only available for 202 patients. Therefore, the statistical analysis of QoL versus LL was performed on 202 patients.

### Survey instruments

#### EORTC-QLQ-C30 and QLQ-H&N43

Health-related QoL was assessed using the European Organisation for Research and Treatment of Cancer Quality of Life Questionnaire Core 30 (EORTC QLQ-C30) in combination with the head and neck cancer–specific module EORTC QLQ-H&N43, both developed by the European Organisation for Research and Treatment of Cancer (EORTC).

The QLQ-C30 is a validated 30-item questionnaire designed to assess general cancer-related QoL across multiple domains, including global health status, five functional scales (physical, role, emotional, cognitive, and social functioning), three symptom scales (fatigue, pain, and nausea and vomiting) [9]. The QLQ-H&N43 is a disease-specific module for patients with head and neck cancer and complements the QLQ-C30 by assessing symptoms and functional impairments particularly relevant to this patient population, such as pain, swallowing difficulties, speech problems, dry mouth, sensory changes, and social eating [10]. The QLQ-C30 has been extensively validated in international cancer populations and has demonstrated good reliability and construct validity. In the original international validation study, all multi-item scales except role functioning met the predefined minimum criterion for internal consistency (Cronbach’s α ≥ 0.70) [9]. Test–retest reliability was also high for most scales, with correlation coefficients ranging from 0.82 to 0.91 for the functional scales and 0.85 for global health status [9]. The EORTC QLQ-H&N43 demonstrated good reliability in an international validation study involving 812 patients with head and neck cancer from 18 countries, with Cronbach’s α > 0.70 in 10 of 12 multi-item scales and ICC > 0.70 in all but two scales, as well as good construct validity and responsiveness to clinical changes [10]. In the present study, all items were scored according to the EORTC scoring manual. Raw scores were linearly transformed to a 0–100 scale, with higher scores representing better functioning or more severe symptoms depending on the scale.

#### Distress thermometer

The validated German adaption of the Distress Thermometer (DT), an 11-point visual analogue scale ranging from 0 (no distress) to 10 (severe distress), was used to quantify the overall level of distress experienced over the past week [11]. Patients with a score of 4 or more on the visual analogue scale are considered to have distress [12, 13]. It has been validated in cancer populations and demonstrated good overall diagnostic accuracy for detecting psychological distress. A meta-analysis of 42 studies involving 14,808 patients reported a pooled sensitivity of 81% and specificity of 72% at the cut-off score of ≥ 4, supporting its use as a brief screening instrument for cancer-related distress [14].

#### Quality and quantity questionnaire

Patient attitudes toward the tradeoff between quality and quantity of life were assessed using the Quality and Quantity Questionnaire (QQ Questionnaire), developed and validated by Stiggelbout et al. [15]. The questionnaire is designed to measure cancer patients’ preferences regarding QoL versus LL, capturing the relative importance they assign to extending survival compared with maintaining or improving health-related QoL. The original QQ questionnaire comprises eight items divided into two preference dimensions: QoL and LL. Patients rate each statement on a 5-point Likert scale, indicating the degree to which they agree or disagree. Higher scores on the quantity or quality scales reflect greater importance placed on LL or QoL, respectively [15]. In this study, a modified 9-item German version of the QQ questionnaire was used. This version has been validated, and demonstrated acceptable reliability (LL subscale (Cronbach’s α = 0.71; QL subscale (Cronbach’s α = 0.59) and validity [16].

### Statistical analysis

For the descriptive and univariable analyses, nominal and ordinal variables were described as absolute and relative frequencies and compared between two groups using Pearson’s chi-square test. For continuous variables, histograms were inspected to check for normal distribution. Normally distributed variables were presented as mean and standard deviation and compared using the t-test. Not normally distributed continuous variables were described as median and 25th and 75th percentiles and compared using the Wilcoxon rank-sum test.

Because the dependent variables, QoL and distress, were assumed to be non-normally distributed, non-parametric methods were applied in the univariable analyses. Associations with continuous variables were assessed using Spearman’s rank correlation coefficient, whereas dichotomous categorical variables were compared using the Wilcoxon rank-sum test. In addition to the univariable analyses, multivariable regression analyses were performed including all variables listed in Table 1, to account for possible correlations between variables. Before conducting the multiple linear regression, the assumption of normality of the regression residuals was tested. Based on the QQ plots for both sets of regression residuals, the residuals were considered to be normally distributed. Variable selection was performed using a forward stepwise procedure. For categorical predictors, regression coefficients were reported relative to the reference category defined automatically by IBM SPSS Statistics.

**Table 1:** Patient and tumor characteristics of the study cohort (*n* = 207)

Parameter		Overall (%)
Sex	Male	146 (70.5)
	Female	61 (29.5)
Tumor location	Nasopharynx	15 (7.2)
	Oropharynx	103 (49.8)
	Hypopharynx	12 (5.8)
	Larynx	52 (25.1)
	Cancer of unknown primary (CUP)	11 (5.3)
	Multiregional tumor	3 (1.4)
	Parotid gland	11 (5.3)
Histology	Squamous cell carcinoma	186 (91.6)
	Adenoid cystic carcinoma	3 (1.5)
	Mucoepidermoid carcinoma	4 (2.0)
	Salivary duct carcinoma	5 (2.5)
	Rhabdomyosarcoma	1 (0.5)
	Adenocarcinoma	3 (1.5)
	Synovial sarcoma	1 (0.5)
	NA	4 (2.0)
p16 status of oropharynx squamous cell carcinoma (OPSCC) (*n* = 79)	p16-positive	61 (7.2)
	p16-negative	18 (22.8)
Union Internationale Contre le Cancer (UICC) tumor stage	I	86 (42)
	II	39 (19)
	III	30 (14.6)
	IVA	35 (17.1)
	IVB	8 (3.9)
	IVC	7 (3.4)
	NA	2 (1.0)
Therapy	Surgery	77 (37.2)
	Surgery + adjuvant radiotherapy	39 (18.8)
	Surgery + adjuvant radiochemotherapy	44 (21.3)
	Definitive radiotherapy	8 (3.9)
	Definitive radiochemotherapy	36 (17.4)
	Palliative radio(chemo)therapy	3 (1.4)
Time in month since diagnosis	Median	32 [16; 59]
Tumor recurrence	Yes	36 (17.4)
	No	171 (82.6)
Current tumour-free status	Yes	184 (88.9)
	No	23 (11.1)
Marital status	Single	21 (10.1)
	Married	122 (58.9)
	Divorced	4 (1.9)
	Widowed	24 (11.6)
	Living with partner	25 (12.1)
	Other	11 (5.3)
Living alone	Yes	61 (29.5)
	No	146 (70.5)
Highest school degree	Lower secondary school leaving certificate	67 (32.5)
	Secondary school leaving certificate	67 (32.5)
	University entrance qualification	15 (7.3)
	A-levels	49 (23.8)
	No school leaving certificate	8 (3.9)
	NA	1 (0.5)
Vocational education	Apprenticeship	101 (49)
	Technical college (e.g., master craftsman school, technical school, vocational academy)	26 (12.6)
	Advanced technical college	9 (4.4)
	University degree	41 (19.9)
	No vocational education	24 (11.7)
	Other vocational education	5 (2.4)
	NA	2 (1.0)
Employment status (*n* = 205)	Employed	49 (23.9)
	Self-employed / freelance	16 (7.8)
	Civil servant	7 (3.4)
	Part-time employed	4 (2.0)
	Not employed	18 (8.8)
	Retired	111 (54.1)
	NA	2 (1.0)
Comorbidities (*n* = 205)	No pre-existing diseases	82 (39.8)
	One pre-existing disease	55 (26.7)
	≥ 2 pre-existing diseases	69 (33.5)
	NA	2 (1.0)

Data were shown as absolute frequency (%) for categorical variables, mean (± standard deviation) for normally distributed continuous variables and median (IQR) for not normally distributed continuous variables; NA: not available

To operationalize patients’ preference for LL versus QoL, a difference score was calculated for each participant by subtracting the mean LL score from the mean QoL score (QoL − LL). Based on this difference score, participants were classified into three preference groups: LL preference (difference < − 0.5), equal preference (difference from − 0.5 to + 0.5), and QoL preference (difference > + 0.5). Differences between the three preference groups were examined using Pearson’s chi-square test for categorical variables, one-way analysis of variance (ANOVA) for normally distributed continuous variables, and the Kruskal–Wallis test for non-normally distributed continuous variables. If the p-value for the overall comparison was statistically significant, pairwise comparisons between all three pairs were performed using chi-square test for categorical variables, t-test for normally distributed continuous variables and the Wilcoxon rank-sum test for not normally distributed continuous variables. Effect sizes were reported as Spearman’s correlation coefficient for correlation analyses. Statistical analyses were performed using IBM SPSS Statistics (Version 31.0; IBM Corp., Armonk, NY, USA). All statistical tests were two-sided, and statistical significance was defined as *p* < 0.05.

## Results

The study cohort comprised 207 patients eligible for analysis. Of these, 70.5% were men. The mean age was 66.2 (± 10.42) years, and the median time since diagnosis was 32 months. The majority of tumors (91.6%) were squamous cell carcinomas, with oropharyngeal carcinomas representing the largest subgroup (49.8%). The most common curative treatment method was surgery alone (37.2%), followed by surgery combined with adjuvant chemoradiation (21.3%) as curative approaches. All demographic and clinical parameters are summarized in Table 1.

**Fig. 1:**
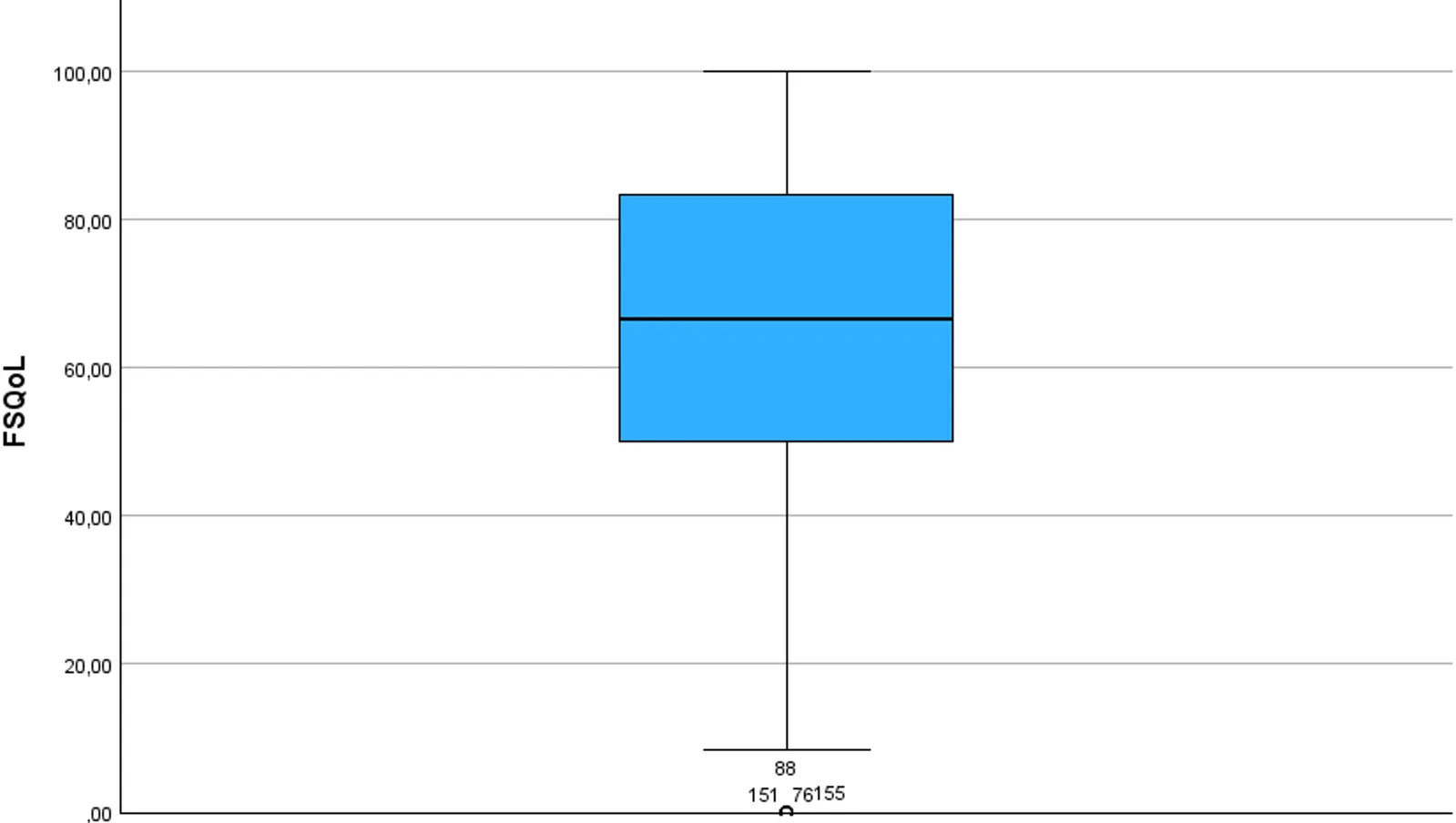
Boxplot of Global Health Status scores measured using the EORTC QLQ-C30 questionnaire. Higher scores indicate better quality of life

The distribution of QoL within the study population was examined using the QLQ-C30 Global Health Status score, expressed on a scale from 0 to 100. As illustrated in Fig. 1, the median score was 66.6 [interquartile range: 50;83.3]. Associations between QoL and potential explanatory variables were first examined using univariable analyses and were subsequently evaluated in a multivariable linear regression model. All variables from Table 1 were included in these analyses, as correlations with QoL could be expected for these predictors.

In the multivariable analysis, higher distress scores were independently associated with lower QoL (b=-3.485, 95% CI [-4.582, -2.387], *p* < 0.001). In addition, compared with the reference employment category, being employed (b = 10.279, 95% CI [1.387, 19.171], *p* = 0.024) and being self-employed (b = 19.327, 95% CI [8.178, 30.477], *p* < 0.001) were independently associated with higher QoL.

When comparing surgical-based approaches (surgery alone or surgery combined with adjuvant therapies) with non-surgical approaches, a significant difference in QoL was identified (*p* = 0.035) with patients treated with surgery-based therapies reporting a better QoL. Similarly, stratifying participants by comorbidity burden, defined as low (none or one pre-existing condition) versus high (two or more pre-existing conditions), revealed a significant difference in Global Health Status scores (*p* = 0.031).

**Fig. 2:**
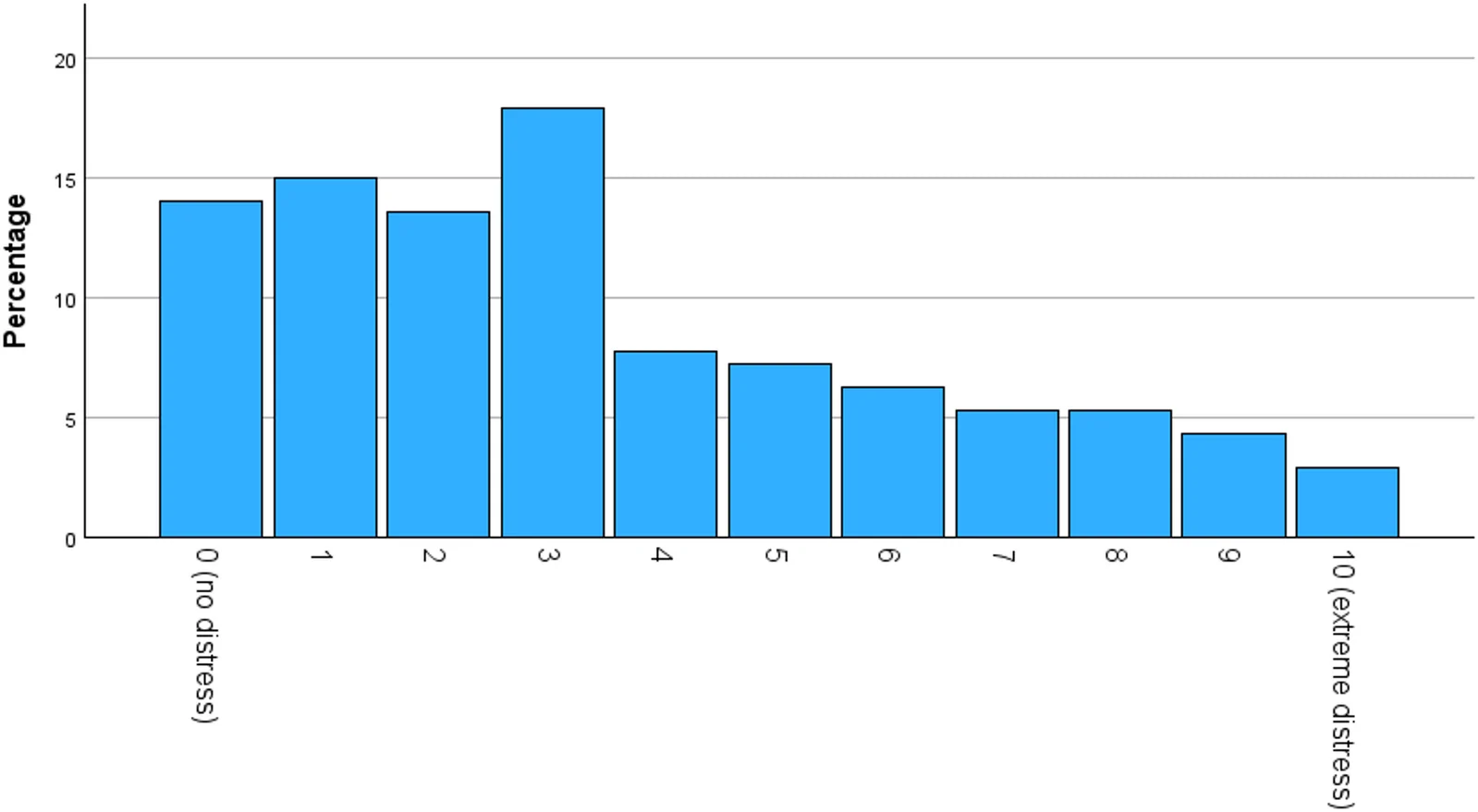
Distribution of distress scores

To identify independent predictors of distress, a second multivariable linear regression analysis was performed with the distress score as the dependent variable. The median distress score was 3 (Fig. 2).

In the multivariable analysis, the following variables were significant independent predictors of distress: sex (b = 0.965, 95% CI [0.043, 1.887], *p* = 0.040), global health status (b = − 0.058, 95% CI [− 0.076, − 0.040], *p* < 0.001), tumor stage UICC stage II (b = 1.178, 95% CI [0.121, 2.234], *p* = 0.029, reference UICC stage I), A-level education (b = − 1.753, 95% CI [− 3.282, − 0.224], *p* = 0.025; reference: secondary school leaving certificate) and definitive chemoradiation (b = − 1.480, 95% CI [− 2.825, − 0.136], *p* = 0.031; reference: surgery) were statistically significant independent predictors of distress.

The central objective of this study was to assess participants’ preferences regarding LL or QoL. Overall, 21.8% of 202 participants expressed a preference for LL, whereas 53.0% favored QoL. The remaining 25.2% were classified as ‘equal’ because their responses did not indicate a clear preference. Detailed results are shown in Table 2.

**Table 2:** Results of the QQ questionnaire (n=202)

	completely disagree	mostly disagree	partly agree	mostly agree	completely agree
**Length of life**					
If a treatment can prolong my life, I will always accept it, whatever the side effects may be.	18 (8.9%)	38 (18.8%)	58 (28.7%)	55 (27.2%)	33 (16.3%)
Even though the treatment is difficult to bear, I would accept it if the chance living longer were increased even by as little as one percent.	14 (6.9%)	24 (11,9%)	41 (20.3%)	65 (32,2%)	58 (28,7%)
If I reached a point during treatment at which I felt like giving up, I would probably manage to find the strength to continue.	13 (6.4%)	26 (12.9%)	47 (23.3%)	72 (35.6%)	44 (21.8%)
In order to live a bit longer I would clutch at any straw.	22 (10.9%)	28 (13.7%)	46 (22.8%)	59 (29.2%)	47 (23.3%)
**Quality of life**					
I will only undergo life-prolonging treatment if I can continue to lead a normal life.	28 (13.9%)	17 (8.4%)	45 (22.3%)	57 (28.2%)	55 (27.2%)
I can imagine some side effects being so bad that I would refuse the treatment, even if that meant a shorter life.	34 (16.8%)	33 (16.3%)	43 (21.3%)	43 (21.3%)	49 (24.3%)
A moment might come at which I would say ‘I have done my best, this is the limit’.	69 (34.2%)	50 (24.8%)	34 (16.8%)	29 (14.4%)	20 (9.9%)
If I had to endure 6 months of intensive treatment in order to live for an extra half year, then I wouldn’t bother.	67 (33.2%)	40 (19.8%)	43 (21.3%)	28 (13.9%)	24 (11.9%)
If it is doubtful whether I can extend my life with a burdensome treatment, then I would rather choose a treatment that places a special emphasis on the quality of life.	10 (5%)	15 (7.4%)	44 (21.8%)	64 (31.7%)	69 (34.2%)

Data were shown as absolute frequency (%)

A significant association between age and preference was observed (*p* = 0.035, Cramer’s V = 0.183), with an increasing inclination toward QoL in older age groups. In pairwise comparisons, a significant difference was observed between the LL and QoL groups (unadjusted *p* = 0.02, Cramer’s V = 0.256). Tumor stage, as defined by the UICC classification, was also significantly associated with patient preference (*p* = 0.032), with preferences appearing to converge as disease stage increased. The proportion favoring LL versus QoL was 10.6% versus 61.2% in stage I, compared with 24.5% versus 49.0% in stage IV. Pairwise comparisons showed significant differences between LL and QoL (unadjusted *p* = 0.007, Cramer’s V = 0.284) and LL and equal preference (unadjusted *p* = 0.041, Cramer’s V = 0.296).

Furthermore, a significant difference was observed when comparing patients treated with surgery alone with those receiving other treatment modalities (*p* = 0.042, Cramer’s V = 0.177). As shown in Table 3, both groups predominantly favored QoL (> 50%). In the dichotomized chi-square analysis, a statistically significant difference was found between LL vs. QoL (*p* = 0.041, Cramer’s V = 0.177) and LL vs. equal preference (unadjusted *p* = 0.014, Cramer’s V = 0.253). Among patients treated with surgery alone who expressed a clear preference (i.e., excluding those with equal preference), 81.1% prioritize QoL compared with 18.9% who prioritized LL, which reflects a more distinct preference than in all other treatment groups (Table 3).

**Table 3:** Association between patient characteristics and outcome groups (long living, equal, and quality of life) in the study cohort (*n* = 202)

Variable	Total *n* = 202	Long living	Equal	Quality of life	*p*-value overall (Cramer’s V)	*p*-value LL vs. QoL (Cramer’s V)	*p*-value LL vs. Equal (Cramer’s V)	*p*-value QoL vs. Equal (Cramer’s V)
Sex					0.6			
Male	142 (70.3)	32 (22.5)	38 (26.8)	72 (50.7)				
Female	60 (29.7)	12 (20)	13 (21.7)	35 (58.3)				
Age group					0.035 (0.183)	0.02 (0.256)	0.27 (0.204)	0.113 (0.195)
< 50	12 (5.9)	5 (41.7)	3 (25)	4 (33.3)				
50–59	34 (16.8)	9 (26.5)	13 (38.2)	12 (35.3)				
60–69	77 (38.1)	20 (26)	16 (20.8)	41 (53.2)				
> 70	79 (39.1)	10 (12.7)	19 (24.1)	50 (63.3)				
Marital status					0.551			
Single	21 (10.4)	3(14.3)	6 (28.6)	12 (57.1)				
Married	119 (58.9)	26 (21.8)	33 (27.7)	60 (50.4)				
Divorced	4 (2)	2 (50)	1 (25)	1 (25)				
Widowed	23 (11.4)	5 (21.7)	5 (21.7)	13 (56.6)				
Living with partner	24 (11.9)	5 (20.8)	2 (8.3)	17 (70.8)				
Others	11 (5.4)	3 (27.3)	4 (36.4)	4 (36.4)				
Living alone					0.324			
Yes	59 (29.2)	10 (16.9)	13 (22)	36 (61)				
No	143 (70.8)	34 (23.8)	38 (26.6)	71 (49.7)				
Highest school degree	*N* = 201				0.183			
Lower secondary school leaving certificate	64 (31.8)	19 (29.7)	19 (29.7)	26 (40.6)				
Secondary school leaving certificate	66 (32.8)	13 (19.7)	16 (24.2)	37 (56.1)				
University entrance qualification	15 (7.5)	0 (0)	5 (33.3)	10 (66.7)				
A-levels	48 (23.9)	11 (22.9)	8 (16.7)	29 (60.4)				
No school leaving certificate	8 (4)	1 (12.5)	3 (37.5)	4 (50)				
Vocational education	*N* = 201				0.351			
Apprenticeship	97 (48.3)	21 (21.6)	28 (28.9)	48 (49.5)				
Technical college (e.g., master craftsman school, technical school, vocational academy)	25 (12.4)	8 (32)	5 (20)	12 (48)				
Advanced technical college	9 (4.5)	2 (22.2)	1 (11.1)	6 (66.6)				
University degree	41 (20.4)	6 (14.6)	9 (22)	26 (63.4)				
No vocational education	24 (11.9)	7 (29.2)	7 (29.2)	10 (41.7)				
Other vocational education	5 (2.5)	0	0	5 (100)				
Employment status	*N* = 200				0.199			
Employed	46 (23)	16 (34.8)	13 (28.3)	17 (37)				
Self-employed / freelance	16 (8)	4 (25)	2 (12.5)	10 (62.5)				
Civil servant	7 (3.5)	2 (28.6)	1 (14.3)	4 (57.1)				
Part-time employed	4 (2)	0	2 (50)	2 (50)				
Not employed	18 (9)	2 (11.1)	7 (38.9)	9 (50)				
Retired	109 (54.5)	20 (18.3)	25 (22.9)	64 (58.7)				
Employment status groups	*N* = 200				0.091			
Working (employed, self-employed / free-lance, civil servant, part-time employed)	73 (36.5)	22 (30.1)	18 (24.7)	33 (45.2)				
Retired + not employed	127 (63.5)	22 (17.3)	32 (25.2)	73 (57.5)				
Localisation					0.810			
Oropharynx	99 (49)	21 (21.2)	27 (27.3)	51 (51.5)				
Others	103 (51)	23 (22.3)	24 (23.3)	56 (54.4)				
UICC					0.032 (0.186)	0.007 (0.284)	0.041 (0.296)	0.589 (0.111)
I	85 (42.5)	9 (10.6)	24 (28.2)	52 (61.2)				
II	39 (19.5)	14 (35.9)	10 (25.6)	15 (38.5)				
III	27 (13.5)	8 (29.6)	4 (14.8)	15 (55.6)				
IV (A-C)	49 (24.5)	12 (24.5)	13 (26.5)	24 (49)				
Therapy					0.042 (0.177)	0.041 (0.166)	0.014 (0.253)	0.414 (0.065)
Only Surgery	77 (38.1)	10 (13)	24 (31.2)	43 (55.8)				
Other	125 (61.9)	34 (27.2)	27 (21.6)	64 (51.2)				
Tumor recurrence								
yes	36 (17.8)	4 (11.1)	10 (27.8)	22 (61.1)	0.229			
no	166 (82.2)	40 (24.1)	41 (24.7)	85 (51.2)				
Current tumour-free status					0.625			
Yes	179 (88.6)	39 (21.8)	47 (26.3)	93 (52.0)				
No	23 (11.4)	5 (21.7)	4 (17.4)	14 (60.9)				
Time in months since diagnosis					0.814			
0–12 months	34 (16.8)	6 (17.6)	9 (26.5)	19 (55.9)				
> 13 months	168 (83.3)	38 (22.6)	42 (25)	88 (52.4)				
Comorbidities	*N* = 201				0.585			
No pre-existing diseases	80 (39,8)	16 (20)	24 (30)	40 (50)				
One pre-existing disease	55 (27,4)	12 (21,8)	15 (27.3)	28 (50.9)				
≥ 2 pre-existing diseases	66 (32,8)	16 (24,2)	12 (18.2)	38 (57.6)				
Distress-Scale					0.320			
< 5	138 (68,3)	33 (23,9)	31 (22.5)	74 (53.6)				
>=5	64 (31,7)	11 (17,2)	20 (31.3)	33 (51.6)				

LL: long living; QoL: Quality of life. Data were shown as absolute frequency (%) for categorical variables, p-value overall, p-value for differences between the three groups (chi-square test for categorical variables); if p-value overall > 0.05, no pairwise comparisons were performed

No significant associations were found between preference and sociodemographic variables including sex, marital status, educational attainment, vocational training, or employment status. Similarly, clinical factors such as tumor location, recurrence, current tumor-free status and comorbidities were not significantly associated with patient preference. Moreover, no significant results were obtained for functional scales (global health status, physical functioning and role functioning (QLQ-C30)) or the multi-item scales (QLQ-H&N43).

## Discussion

Despite medical advances, patients with head and neck tumors face a substantial burden of functional symptoms related to tumor location, such as dysphagia, airway obstruction, or mucositis, as well as physical and psychological symptoms, like fatigue, lack of appetite, pain, and anxiety [17].

Patients who have been diagnosed with cancer and have undergone therapy have likely reflected on whether they would choose treatment despite its side effects. The present study aimed to identify individual preferences regarding LL versus QoL and to investigate whether these preferences are associated with potential predictors.

Several studies have reported impaired QoL among HNC survivors [18, 19]. Interestingly, the present cohort reported low levels of distress and a relatively high overall quality of life. These findings suggest that, despite the challenges associated with head and neck cancer and its treatment, the HNC survivors in this cohort experienced relatively low psychological burden and favorable self-reported quality of life. The median QoL score in our study population was 66.6, with lower distress levels, employment, surgery-based treatment approaches, and lower comorbidity burden being associated with higher QoL. Other studies have demonstrated that physical limitations, such as dysphagia and voice problems, are significantly associated with worse QoL in head and neck cancer survivors [20]. Impaired QoL is of particular relevance, as QoL may itself be associated with prognosis. In several studies, poor baseline QoL scores have been linked to reduced survival rates in patients with HNC [6, 21]. However, this association should be interpreted with caution, as poor QoL may indicate advanced disease, and higher disease stage is likely the primary reason for poorer survival rather than QoL itself.

A central finding of our study is the association between distress and QoL. Distress emerged as a significant predictor of QoL in both univariable and multivariable analyses, with higher distress levels associated with lower global health status. This is in line with existing literature demonstrating that psychological burden is one of the most important determinants of patient-reported outcomes in cancer populations. For example, Hinz et al. showed that cancer patients have an almost twofold higher risk of psychological distress than the general population, although younger patients were at greater relative risk compared with their respective reference group [22]. A recent study demonstrated that the three most frequently reported symptoms in long-term HNC survivors were tiredness, anxiety and drowsiness, whereas the three least frequently reported symptoms were constipation, vomiting and nausea [23]. These findings highlight the importance of routine distress screening and psychosocial support as integral components of oncological care.

Our analysis further showed that female sex and higher tumor stage were independently associated with higher levels of distress while higher educational level and definitive radiochemotherapy were associated with lower distress. Prior studies indicated that women are more vulnerable to psychological burden [24, 25] which underscores the importance of gender-sensitive approaches in clinical care and patient communication. Interestingly, higher educational attainment was associated with lower distress, likely mediated by greater health literacy and access to coping resources. Specifically, patients with higher educational backgrounds are often better equipped to comprehend complex medical protocols, treatment side effects, and long-term prognoses. This increased health literacy may reduce fear of the unknown and may foster a greater sense of personal control. Furthermore, these individuals often possess stronger socio-economic safety nets and superior problem-solving skills, allowing them to effectively mobilize practical, emotional, and financial resources during cancer care.

Notably, patients who received radiochemotherapy reported lower distress in the analyzed patient cohort, whereas those treated with surgery alone showed better quality of life. This pattern is unexpected, as distress and quality of life are usually closely linked and would be expected to move in the same direction. One possible explanation is that the two constructs capture different aspects of the patient experience: distress may reflect psychological burden and coping, while quality of life is more strongly shaped by functional outcomes such as swallowing, speech, and treatment-related late effects. Irrespective of the underlying mechanism, these findings suggest that, in certain subgroups of patients with head and neck cancer, lower distress does not automatically translate into better quality of life, which underlines the importance of psychosocial care that addresses patients’ overall well-being, regardless of treatment modality.

It has been widely demonstrated that psychological support can improve perceived QoL in patients with cancer [26]. Of particular interest are recent findings showing that digital psychological support services also have a positive impact on QoL [27, 28]. As older patients become increasingly comfortable with digital tools, even more patients could benefit from such support in the future.

Extending life expectancy in HNC patients often necessitates a more radical treatment approach which can result in physical impairments [20]. Consequently, patients are frequently confronted with the question of whether to pursue longer survival at any cost or to prioritize the best possible QoL, even if this means not exhausting all available treatment options.

In clinical practice, there are often discrepancies between the treatment goals of physicians, who traditionally focus more on oncological endpoints, such as overall survival, and the subjective priorities of patients, who frequently place greater value on quality of daily life, freedom from pain, the ability to communicate, and emotional stability. Prolongation of survival therefore cannot be regarded as the only indicator of therapeutic success. Our findings show that 53% of respondents clearly favored QoL representing more than twice the proportion of patients who prioritized life prolongation. In addition, an age-related trend became apparent, with preferences shifting progressively toward QoL with increasing age. A study by Meropol et al. [29], involving patients with various advanced cancers, showed that a preference towards QoL was associated with older age, male sex, and higher educational level. Patients with a QoL preference also reported lower levels of cancer-related distress [29]. Further associations between a preference for QoL and sociodemographic factors are difficult to identify, which may explain the limited research available on this topic. This suggests that preferences may be shaped more by individual values than by objective characteristics. Interestingly, based on their questionnaire responses, a larger proportion of patients in our study were classified into the intermediate group (“equal”; 25.2%) than into the LL group (21.8%). In the study by Meropol et al., as many as 55% of patients could not be assigned to a distinct preference group, which the authors attributed to the difficulty patients face in clearly distinguishing between LL and QoL in a hypothetical setting [29]. It seems natural that patients’ opinions lie along a continuum and that there is a degree of indecision and that, in a hypothetical scenario, patients tend to take a relatively neutral stance to keep their options open. A narrative review highlights that the dichotomization of LL and QoL may be distressing for patients and that most treatment decisions fall somewhere in between these two extremes. Nevertheless, LL and QoL are often mistakenly seen as competing, non-coexisting opposites [30].

Trade-off scenarios are an established method for measuring subjective priorities. A study published in 2020 attempted to evaluate the relationship between survival time benefit and QoL benefit as economic goods. The key question was whether these two benefits could be defined as perfect substitutes or as perfect complements. This study showed that cancer patients place similar importance on survival time, QoL, and their willingness to accept adverse events during cancer treatment [31].

The present analysis, together with current evidence, underscores the importance of involving patients in treatment planning at an early stage and in a structured manner, particularly when addressing the trade-off between LL and QoL. Several studies have demonstrated that open, value-based communication about treatment goals, symptom management, and individual preferences can improve the treatment experience, increase acceptance of treatment decisions, and ultimately improve subjective well-being [32].

Our study has several limitations. Firstly, the cross-sectional design limits causal inference and the assessment of changes over time. Secondly, the relatively small sample size within certain subgroups may have limited statistical power, and resulted in unstable estimates, particularly in the multivariable analyses. Thirdly, classifying participants into preference groups based on mean difference scores may have reduced variability in responses and thereby resulted in some loss of information, although this is a commonly used approach. Finally, the data were self-reported, and patients completed the questionnaires as part of their cancer aftercare which may have influenced their current mood, distress, and tendency to respond in a socially desirable manner.

Nevertheless, a key strength of this study is that it shows that patients’ preferences are largely individual and not predetermined. The clinical implication is that preferences must be actively explored in shared decision-making and cannot be reliably deduced from clinical parameters.

## Conclusion

In conclusion, this study demonstrates that most head and neck cancer survivors prioritize QoL​ over LL, while a substantial proportion remain undecided, reflecting the complexity of this trade-off. Preferences were not strongly associated with most clinical or sociodemographic variables, suggesting that they are primarily shaped by individual values rather than objective characteristics. Notably, the observed shift toward QoL with increasing age highlights the need for age-sensitive communication and individualized care strategies. Overall, these findings indicate that treatment decisions in head and neck cancer should not be guided solely by clinical parameters, but should incorporate structured, patient-centered discussions about individual preferences, particularly regarding the balance between prolonging life and maintaining QoL.

## Data Availability

The datasets generated during and/or analysed during the current study are available from the corresponding author on reasonable request.
